# Depression and Anxiety in Old Age during the COVID-19 Pandemic: A Comparative Study of Individuals at Cardiovascular Risk and the General Population

**DOI:** 10.3390/ijerph20042975

**Published:** 2023-02-08

**Authors:** Sina K. Gerhards, Melanie Luppa, Susanne Röhr, Alexander Pabst, Alexander Bauer, Thomas Frankhänel, Juliane Döhring, Catharina Escales, Isabel Renate Zöllinger, Anke Oey, Christian Brettschneider, Birgitt Wiese, Wolfgang Hoffmann, Jochen Gensichen, Hans-Helmut König, Thomas Frese, Jochen René Thyrian, Hanna Kaduszkiewicz, Steffi G. Riedel-Heller

**Affiliations:** 1 Institute of Social Medicine, Occupational Health and Public Health (ISAP), University of Leipzig, 04103 Leipzig, Germany; 2 Health and Ageing Research Team (HART), School of Psychology, Massey University, Palmerston North 4474, New Zealand; 3 Institute of General Practice and Family Medicine, Martin-Luther-University Halle-Wittenberg, 06112 Halle (Saale), Germany; 4 Institute of General Practice, University of Kiel, 24105 Kiel, Germany; 5 Institute of General Practice/Family Medicine, University Hospital of LMU Munich, 80336 Munich, Germany; 6 Institute for General Practice, Work Group Medical Statistics and IT-Infrastructure, Hannover Medical School, 30625 Hannover, Germany; 7 Department of Health Economics and Health Services Research, University Medical Center Hamburg-Eppendorf, 20246 Hamburg, Germany; 8 Institute for Community Medicine, University Medicine Greifswald, 17475 Greifswald, Germany; 9 German Center for Neurodegenerative Diseases (DZNE), Site Rostock/Greifswald, 17489 Greifswald, Germany

**Keywords:** depressive symptoms, anxiety, cardiovascular risk, old age, COVID-19 pandemic

## Abstract

Our study aims to examine the associations of sociodemographic factors, social support, resilience, and perceptions of the COVID-19 pandemic with late-life depression and anxiety symptoms in a cardiovascular risk group and a matched sample from the German general population during the beginning of the pandemic and draw a comparison regarding psychosocial characteristics. Data of *n* = 1236 participants (aged 64–81 years) were analyzed, with *n* = 618 participants showing a cardiovascular risk profile, and *n* = 618 participants from the general population. The cardiovascular risk sample had slightly higher levels of depressive symptoms and felt more threatened by the virus due to pre-existing conditions. In the cardiovascular risk group, social support was associated with less depressive and anxiety symptoms. In the general population, high social support was associated with less depressive symptoms. Experiencing high levels of worries due to COVID-19 was associated with more anxiety in the general population. Resilience was associated with less depressive and anxiety symptoms in both groups. Compared to the general population, the cardiovascular risk group showed slightly higher levels of depressive symptomatology even at the beginning of the pandemic and may be supported by addressing perceived social support and resilience in prevention programs targeting mental health.

## 1. Introduction

The SARS-CoV-2 virus outbreak in 2019 heavily impacted the daily life of people all over the world. In order to curb the virus outbreak, the German government, like administrations in many other countries, ordered a nationwide lockdown. There is evidence from previous pandemics, for example, the SARS pandemic in 2003/2004, and from first investigations during the COVID-19 pandemic, that lockdowns, quarantine, and social distancing can have serious, negative consequences for the social and mental health of the affected population [1,2,3].

Particularly for high-risk groups for a severe and lethal course of COVID-19 disease, the pandemic can be a strong psychological burden. This includes people older than 60 years in general, and people with pre-existing health conditions in particular, with cardiovascular diseases as the most common pre-condition among deceased patients with COVID-19 [4]. People with cardiovascular diseases and with cardiovascular risk factors may have a higher risk of a severe course of disease since those pre-conditions can aggravate hyper-inflammatory processes [5,6]. There is evidence from previous studies that cardiovascular risk factors such as hypercholesterolemia, hypertension, physical inactivity, and overweight are associated with depressive and anxiety disorder symptomatology [7,8,9]. Taking the risk of a severe course of disease and the higher risk for anxiety and depression symptoms together, the investigated cardiovascular risk group may be more psychologically burdened compared to the general old age population, and there are studies showing higher psychological burden in terms of depression and anxiety in people with pre-existing conditions [10,11], with one study by Deimel et al. (2022) specifically comparing depressive and anxiety symptoms during the COVID-19 pandemic in COVID-19-high-risk versus low-risk patients. Results showed that the depressive and anxiety symptoms of the high-risk group were higher compared to the low-risk-group. The categorization of high and low risk was conducted based on a broader risk score system including medical comorbidities such as cardiovascular diseases, cancer, diabetes mellitus, and others, and the study sample was of young adult age [10].

Social distancing may have increased the feeling of not feeling socially integrated, impacting especially those who took measures extremely seriously due to pre-existing conditions [12]. High perceived social support may have a positive effect on the individual’s psychological health during the pandemic, e.g., by buffering stress levels [13]. Levkovich et al. [14] found that high social support is associated with lower depression levels during the pandemic in an older Israeli sample. Özmete and Pak [15] found that higher social support was associated with less anxiety during the pandemic. In regard to resilience, the first research in this field suggests that resilience may play a major role in coping in an adaptive and healthy way in old age, with high resilience levels being associated with less anxiety and less depressive symptoms in the general population [16,17,18,19,20].

To our knowledge, there has not been an investigation of social support, resilience, and their associations with the mental health factors of depressive and anxiety symptoms in (a) a cardiovascular risk sample, and (b) in a sample that is also of old age during the COVID-19 pandemic in Germany.

It is important to identify factors associated with depressive and anxiety symptoms during the pandemic to adequately support people at risk for a severe and lethal course of COVID-19 disease and to decrease mental distress, as mental distress is again associated with a higher risk of a severe course of disease through inflammatory mechanisms [21].

Against this background, we aim to examine differences between the cardiovascular risk group and the general population in mental distress in terms of anxiety and depression, and potential protective factors such as social support and resilience. Moreover, we aim to investigate the association of sociodemographic factors, worries about the COVID-19 pandemic, being supportive of governmental measures, as well as social support and resilience with depressive and anxiety symptomatology in the cardiovascular risk sample and compare it with those of the general population.

## 2. Methods

### 2.1. Procedure and Participants

The current cross-sectional study analyses data from two data sources: (1) a cardiovascular risk sample originated from the AgeWell.de Intervention study [22,23], and (2) a sample of a representative survey conducted by a leading social research institute in Germany called USUMA GmbH, which collected data in the German general population of *n* = 1005 people (aged ≥ 65) by conducting telephone interviews in April 2020 [16].

The sampling was conducted via multi-stage random digital dialing drawn from a sample base of the Association of German Market and Social Research Agency’s (ADM). Telephone numbers were drawn in proportion to the German population structure. The inclusion criteria was an age of 65 years and above [16].

For the sample of the AgeWell.de Intervention with cardiovascular risk profiles, data were collected closely after the first lockdown from April 2020 to July 2020 in the form of a paper–pencil-based questionnaire. Participants were currently enrolled in the AgeWell.de study [22,23] and asked to take part in an additional COVID-19 pandemic-related survey.

The CAIDE (Cardiovascular Risk Factors, Aging, and Incidence of Dementia) score contains information on age, education, gender, hypertension, hypercholesterolemia, obesity, and physical inactivity. Participants of the AgeWell.de Study had a cardiovascular risk score (CAIDE score) of ≥9, indicating an elevated cardiovascular risk profile that is, for example, associated with late-life dementia [24]. Another inclusion criterion was an age of 60 to 77 years. The sample of participants who filled out the COVID-19 questionnaire consisted of *n* = 874 participants with a mean age of 71.91 (*SD* = 4.36). Thus, the AgeWell.de sample represents a high-risk sample due to its old age and elevated cardiovascular risk profiles. Exclusion criteria were conditions that affect the safe participation in the AgeWell.de Study, such as malignant diseases/fatal diseases, severe clinical depression, symptomatic cardiovascular disease, and revascularization within the past year as evaluated by the general practitioner. Moreover, not being able to speak and read German; severe loss of hearing, vision, or ability to communicate; as well as severe mobility impairment; a previous dementia diagnoses/suspected dementia by the general practitioner; or the participation in another study trial were reasons for exclusion [22,23].

**Figure 1:**
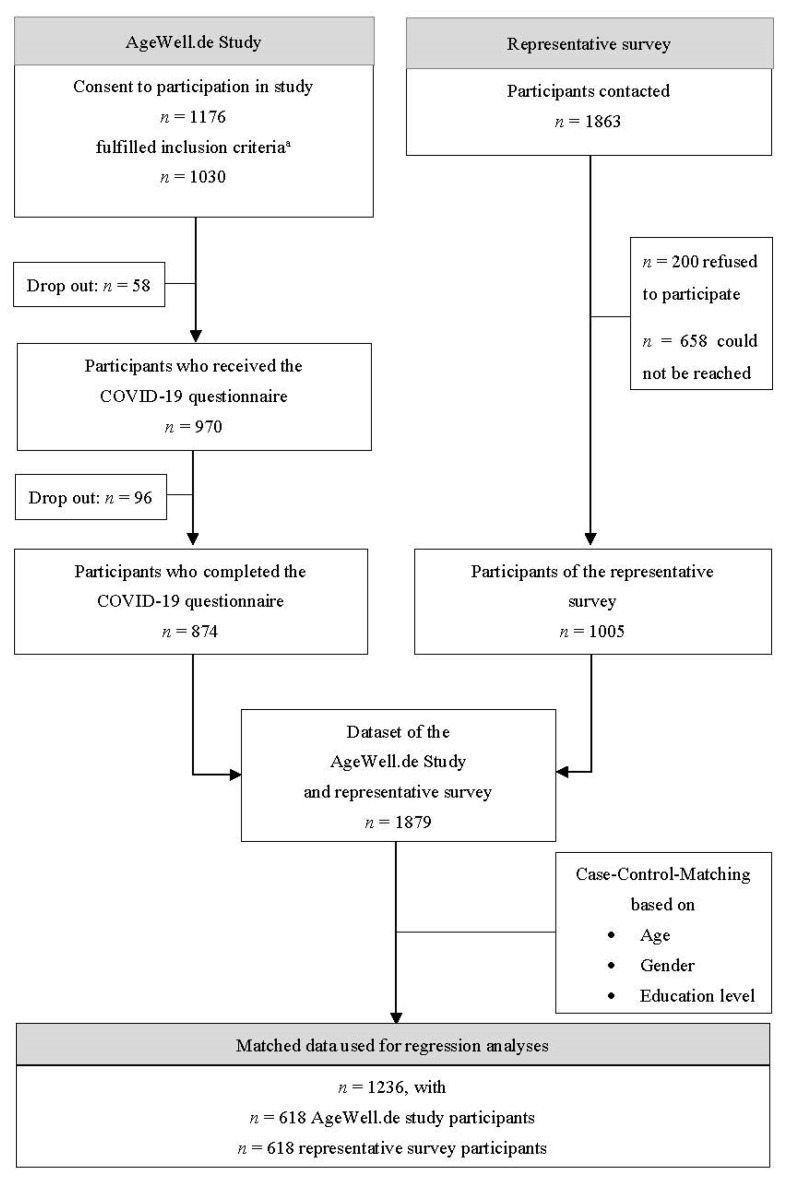
Inclusion flowchart. Notes. ^a^ See AgeWell.de study protocol for more detailed information on the inclusion criteria [22].

More details on the data collection can be retrieved from the inclusion flowchart (Figure 1) and from Röhr et al. [16] for the representative study, and from Zülke et al. [22] and Röhr et al. [23] for the AgeWell.de study. Both groups filled out the same questionnaires that are described in detail below.

### 2.2. Measures

#### 2.2.1. Independent Variables

Standardized questions assessed sociodemographic characteristics of the current sample including age (years), gender (male/female), education (categorized according to the Comparative Analysis of Social Mobility in Industry Nations/CASMIN classification: low/middle/high), marital status (married/with partner, divorced/single, widowed), and living situation (alone, with someone).

Participants also answered questions regarding their personal life situation during the COVID-19 pandemic and their attitudes towards it. Items were answered on a five-point Likert Scale (0 to 4) and assessed the extent of worries about COVID-19 (“I am worried because of the Coronavirus”), the extent of perceived personal threat due to pre-existing medical conditions (“I am threatened by the virus due to pre-existing medical conditions”), and the extent of supportiveness of the governmental measures to curb the virus outbreak (“I fully support the governmental measures”).

Perceived social support was assessed using the ENRICHD social support inventory (ESSI) [25]. Five items can be rated on a five-point Likert scale (“never” to “always”, 1 to 5, respectively), with higher scores indicating higher perceived social support. Sum scores were calculated. The scale has shown good psychometric properties in previous studies [25,26].

The Brief Resilience Scale [27] assesses the ability to bounce back from stressful events using six items that can be rated on a five-point Likert Scale (“fully agree” to “fully disagree”, 1 to 5, respectively). To minimize response bias in the form of social desirability, two items are worded positively and two negatively. Mean scores were calculated. In line with Chmitorz et al. [28], we adjusted for a method factor.

#### 2.2.2. Dependent Variables

The anxiety and depression subscale of the Brief Symptom Inventory (BSI-18) [29] measured the extent of depression and anxiety symptomatology consisting of six items, each rated on a five-point Likert scale (“not at all” to “very much”, 0 to 4, respectively). Sum scores were calculated. The BSI-18 subscales for anxiety and depression of the German version shows good psychometric properties [29].

### 2.3. Data Analysis

Data processing and statistical analyses were conducted using IBM SPSS Statistics, Version 25 [30], and STATA 16.0 SE [31]. Descriptive results were shown as frequencies and percentages or means and standard deviations. Group differences were assessed calculating chi-square tests, *t*-tests, and Mann–Whitney *U*-tests, as appropriate.

Participants of both studies were matched using case-control matching based on the key sociodemographic variables of age, gender, and educational level (tolerance level: 1–0–0), tolerating a one-year age difference in matched participants but none in gender and education to establish better comparability. We matched data based on those variables to increase comparability by ensuring similar numbers in the potential confounders of age, gender, and education in both of the samples. This resulted in a final sample of *n* = 1236, with *n* = 618 for each group (see inclusion flowchart in Figure 1). We calculated multivariate linear regression models to assess the association of sociodemographic factors, worries due to COVID-19, the degree of supportiveness of governmental measures, perceived social support, and resilience with depressive and anxiety symptomatology. The regression models included metric variables except from the following nominal or categorial variables: gender (male with reference to female), education as categorized by the Comparative Analysis of Social Mobility in Industrial Nations/CASMIN classification (middle and high with reference to low), marital status (married/with partner, widowed with reference to single/divorced), and living situation (living with someone with reference to living alone). According to Hayes and Cai [32], we calculated robust standard errors with the HC-3 method. We report standardized beta (*β*) coefficients to allow for a direct comparison of coefficients between models and performed omnibus F-tests for all categorical independent variables in the models.

## 3. Results

### 3.1. Sample Characteristics and Differences in Psychosocial Variables

Out of *n* = 1236 participants, *n* = 6 (0.5%) were infected with the SARS-CoV-2 virus at the time of the assessment, with *n* = 4 (0.6%) being from the cardiovascular risk sample and *n* = 2 (0.3%) from the general population sample. Sociodemographic characteristics, perception and attitude towards the COVID-19 pandemic, and psychosocial characteristics of the sample (*n* = 1236) divided by subsamples can be seen in Table 1. Since we matched the samples based on age, gender, and education, there was no significant difference between groups regarding those characteristics. There was a significant difference in marital status between groups (*x*^2^ = 10.28, *p* = 0.006), with individuals of the cardiovascular risk group being less often single or divorced (23.3% vs. 16.5%) and more often married or with a partner (57.4% vs. 65.2%). Compared to the representative group, the cardiovascular risk group experienced a higher threat due to pre-existing conditions (*U* = 219567.0, *p* < 0.001) and was slightly less supportive of the governmental measures (*U* = 172071.0, *p* < 0.001). Participants with a cardiovascular risk profile showed slightly higher levels of depressive symptoms compared to the general population sample (*t* = −2.89, *p* = 0.004). There were no significant differences in terms of worries, anxiety symptoms, resilience, and perceived social support between groups.

### 3.2. Predicting Mental Health Factors in the Cardiovascular Risk Sample

The multivariate regression model explained 25.7% of variance in depressive symptoms in the cardiovascular risk sample (see Table 2). Perceived resilience had the highest effect, with higher perceived resilience being associated with fewer depressive symptoms (*β* = −0.267, *p* < 0.001), closely followed by perceived social support with higher perceived social support being associated with fewer depressive symptoms (*β* = −0.269, *p* < 0.001).

The multivariate regression model predicting anxiety symptoms in the cardiovascular risk sample explained 22.5% of variance in anxiety symptomatology (see Table 2). High perceived social support (*β* = −0.107, *p* = 0.015) and resilience (*β* = −0.368, *p* < 0.001) were associated with fewer anxiety symptoms. Higher perceived threat due to pre-existing medical conditions was associated with more anxiety (*β* = −0.090, *p* = 0.038). Education was a significant predictor of anxiety (*F*(2) = 4.64, *p* = 0.010), with participants with a high educational level showing higher levels of anxiety compared to participants with a low educational level (*β* = −0.125, *p* = 0.015).

### 3.3. Predicting Mental Health Factors in the General Population

The multivariate model of depressive symptoms in the general population explained 23.5% of variance in depressive symptomatology (see Table 3). Social support had the highest effect, with participants perceiving high social support being less depressive (*β* = −0.220, *p* < 0.001). Moreover, high resilience was associated with less depressive symptoms (*β* = −0.185, *p* < 0.001). Marital status was a significant predictor of depression (*F*(2) = 4.80, *p* = 009), with participants who were widowed showing higher depressive levels compared to single or divorced participants (*β* = 0.141, *p* = 0.017). Males showed small but significantly higher depressive levels compared to women (*β* = −0.079, *p* = 0.045).

The multivariate regression model predicting anxiety levels explained 17.9% of variance (see Table 3). Perceiving higher levels of worries about the virus were associated with higher anxiety levels (*β* = −0.121, *p* = 0.005). Participants who perceived high levels of resilience showed less anxiety symptoms (*β* = −0.197, *p* = 0.001). Education was a significant predictor of anxiety (*F*(2) = 3.50, *p* = 0.031) with no differences between high and medium educational levels compared to a low educational level. The statistical significance was derived from using a medium educational level as reference. Participants with high education level showed higher levels of anxiety compared to participants with a medium educational level (*β* = −0.108, *p* = 0.009).

## 4. Discussion

We aimed to examine differences between an old age cardiovascular risk sample and the general old age population in Germany regarding the mental health factors anxiety and depressive symptoms as well as the potential protective factors social support and resilience. Furthermore, the aim was to identify determinants of anxiety and depressive symptoms in each group and compare them with regard to specific determinants.

### 4.1. Differences in Psychosocial Characteristics between the Older Cardiovascular Risk Group and the General Population

Participants with a cardiovascular risk profile showed slightly higher levels of depressive symptoms compared to the general population. This is in line with previous research indicating that individuals with cardiovascular risk factors and, generally, chronic medical conditions have a higher risk for developing depressive symptoms [10,11]. Nevertheless, the difference between the cardiovascular risk sample and the general population was rather small. This could be due to the fact that data were collected at the beginning of the pandemic, right after the first lockdown in 2020, and the potentially psychologically burdening event was rather new. Cardiovascular risk factors may have worsened during quarantine measures since quarantine was often associated with less physical activity and sometimes unhealthy diets [33]. This could strengthen the interpretation that we may have to expect an increase in depressive symptomatology over the course of the pandemic in the old age cardiovascular risk group and the importance of focusing on this topic in further research.

Moreover, the cardiovascular risk group felt more often threatened by the virus due to medical preconditions compared with the general population, and this again is associated with higher anxiety symptoms, potentially putting this group at risk for higher anxiety symptoms over the course of the pandemic as well. On the other hand, the results also showed that the cardiovascular risk group is equipped with resources, such as social support and resilience that buffer the potential mental distress due to the pandemic. Studies should investigate symptoms of depression and anxiety throughout the pandemic to clarify the development of depressive and anxiety symptomatology.

We see that both the cardiovascular risk sample and the general population sample were very supportive of the governmental measures to curb the virus, with average answers indicating high support. Individuals of the general population sample showed even higher support for the governmental decisions compared to the cardiovascular risk group. However, we do not know why the cardiovascular risk group was less supportive. One reason might be that governmental measures were associated with worries about more difficult access to the healthcare infrastructure that may be more important for people with cardiovascular factors. However, the difference in supportiveness was small, and both groups showed rather high support.

We did not see significant differences in perceived social support or resilience between groups, showing that the sample with the cardiovascular risk profile had a comparable perception of social support and resilience and was not particularly vulnerable regarding these resources during the first lockdown. In regard to social support, this would be contrary to the assumption of Wong et al. [12], namely, that especially those with preexisting conditions may be negatively influenced by the pandemic and the associated social distancing, as they feel particularly lonely and not socially integrated. Nevertheless, it is also important to consider that these data represent the situation during the first lockdown and do not illustrate the psychosocial condition of the high-risk group over the course of the pandemic.

### 4.2. Determinants of Anxiety and Depression Symptoms in the Cardiovascular Risk Group Compared to the General Population

When taking a look at the determinants of depression and anxiety in the cardiovascular risk sample, we see that the results support the assumption that social support and resilience can be resources that buffer the effect of a stressful environment on depression and anxiety symptoms. We found that social support is associated with fewer depressive symptoms, indicating that social support can have a protective value against developing depressive symptomatology. This is in line with previous research, e.g., the investigation of Grey et al. [34], who found that higher social support is associated with fewer depressive symptoms in young and middle-aged adults. Furthermore, results are in line with the investigation of an older Turkish sample that showed an association of perceived social support and anxiety, with high social support being associated with fewer anxiety symptoms during the pandemic [15]. Our results suggest that social support shows this protective effect against depressive and anxiety symptoms in the German cardiovascular risk group as well. We do not see the association of social support and anxiety in the general population group. However, we do see the same trend. This may indicate that social support is more important for mental health in terms of anxiety symptoms when an individual is not in their best health and should be taken in the focus of interventions to reduce anxiety in the cardiovascular risk group. This seems especially relevant since social activities decreased during the pandemic (e.g., [35]). Interventions should focus on ways to increase the feeling of social connectedness while still following social distancing measures, e.g., by scheduling more phone and video calls and supporting those who are not that experienced in using this technology.

Our study results regarding resilience are in line with previous research that showed an association between resilience and depressive and anxiety symptoms, with higher resilience levels being associated with lower depressive and lower anxiety symptoms [16,17]. We see this effect in both the cardiovascular risk group and the general population, with even higher effects in the cardiovascular risk group stressing the importance of targeting resilience in both groups and especially the group with cardiovascular predispositions and associated high perceived threat by the virus.

Interestingly, we see that higher levels of worries are associated with more anxiety in the general population but not in the cardiovascular risk group. We know from previous research that the content of worries differs between depressive and anxiety disorders. Some studies differentiate between depressive worries such as worries associated with a lack of self-confidence and negative views about the future and anxious worries such as worries about a lack of control [36]. For our study results, this could mean that the general population may show more anxious worries. Since the current investigation was done in the beginning of the pandemic where we had little information on the nature of the virus and the course of the pandemic, the results may illustrate what is a natural reaction to a stressful event with an uncertain course in the general population. In the cardiovascular risk group, there was no such connection of worries and anxiety levels, but we do see a trend of worries being associated with depressive symptoms. Certainly, we need to be careful with interpretations due to the non-significance of this association, but this could hint to a more depressive worry content in the cardiovascular risk group. Taken together with the higher depressive levels of the cardiovascular risk group compared to the general population, depressive symptoms should be monitored closely to prevent higher depressive levels over the course of the pandemic.

### 4.3. Strengths and Limitations

While the study has many strengths such as the large sample size and the access to the specific group of people with an elevated cardiovascular risk profile and the general population sample, there are also some limiting factors that need to be taken into account. The current study is a cross-sectional study, and thus we cannot draw causal conclusions of the effects. There was a need to keep the questionnaire compact and short since the participants of the AgeWell.de Study with cardiovascular risk profiles already filled out a large number of questionnaires and completed interviews, and there was a time limit for the telephone interviews for the representative survey. This is why we did not assess additional information on mental and physical health that could potentially also influence mental health factors, such as, for example, diagnoses or the subjective health status.

## 5. Conclusions

Compared to the German general population, the old aged cardiovascular risk group shows slightly higher levels of depressive symptomatology, even at the beginning of the pandemic. Perceived social support and resilience may represent important protective factors against the negative consequences of the pandemic on mental health, with even stronger associations in individuals with cardiovascular risk profiles. Targeting these factors in intervention programs could contribute to maintaining good mental health of the old age population and especially the high-risk groups over the course of the pandemic or other stressful health-related events.

## Figures and Tables

**Table 1:** Sociodemographic characteristics, perception, and attitude towards the COVID-19 pandemic and psychosocial variables divided by population (*n* = 1236).

	Representative Survey Sample (*n* = 618)	Cardiovascular Risk Sample (*n* = 618)	Group Differences (*p*-Value)
Sociodemographic characteristics of the matched sample			
Age; *M* (*SD*)	71.99 (4.36)	71.94 (4.38)	0.811
Gender; *n* (%)			1.000
Female	358 (57.9)	358 (57.9)	
Male	260 (42.1)	260 (42.1)	
Education level; *n* (%)			1.000
Low	159 (25.7)	159 (25.7)	
Middle	275 (44.5)	275 (44.5)	
High	184 (29.8)	184 (29.8)	
Marital Status; *n* (%)			0.006
Single/divorced	144 (23.3)	102 (16.5)	
Married/with partner	355 (57.4)	403 (65.2)	
Widowed	117 (18.9)	113 (18.3)	
Living situation; *n* (%)			0.050
Living alone	229 (37.1)	196 (31.7)	
Living with someone	389 (62.9)	421 (68.1)	
Cardiovascular risk score	10.13 (1.24, 9–14)		
*M* (*SD*, range)			
Systolic blood pressure > 140 mmHg; *n* (%)		539 (87.2)	
Body mass index > 30 kg/m^2^; *n* (%)		360 (58.3)	
Total cholesterol > 6.5 mmol/L; *n* (%)		353 (57.1)	
Physical activity less than 2 time/week for at least 30 min; *n* (%)		450 (72.8)	
Perception and attitude towards the COVID-19 pandemic			
Being worried; *M (SD)*	2.54 (1.35)	2.58 (1.10)	0.849
Perceived threat due to pre-existing conditions; *M (SD)*	1.88 (1.59)	2.37 (1.34)	<0.001
Being supportive of the governmental measures; *M (SD)*	3.60 (0.82)	3.49 (0.84)	0.001
Psychosocial variables			
Perceived social support; *M (SD)*	21.50 (3.76)	21.60 (3.88)	0.442
Resilience; *M(SD)*	2.62 (0.68)	2.57 (0.78)	0.298
Depressive symptoms; *M (SD)*	1.28 (1.86)	1.65 (2.52)	0.004
Anxiety symptoms; *M (SD)*	1.55 (1.98)	1.56 (2.36)	0.934

Notes. Missing values: general population: marital status: *n* = 2 (0.3%), being worried, perceived threat, being supportive: *n* = 1 (.2%), social support: *n* = 17 (2.7%), resilience: *n* = 25 (4.1%); depressive symptoms: *n* = 10 (1.6%), anxiety symptoms: *n* = 8 (1.3%); Cardiovascular Risk Group: living situation: *n* = 1 (0.2%), being worried: *n* = 12 (2.0%), perceived threat: *n* = 13 (2.1%), being supportive: *n* = 8 (1.3%), social support: *n* = 24 (3.9%), resilience: *n* = 18 (2.9%); depressive symptoms: *n* = 23 (3.7%), anxiety symptoms: *n* = 21 (3.4%); group differences: resilience, depressive symptoms, anxiety symptoms = *t*-test; gender, education, marital status, living situation = chi-square test; age, social support, being worried, perceived threat due to preexisting condition, being supportive = Mann–Whitney *U*-test.

**Table 2:** Multivariate regression model predicting depression and anxiety symptoms in a cardiovascular risk sample during the COVID-19 pandemic.

	Depressive Symptoms		Anxiety Symptoms	
	*β*	*p*-Value	*β*	*p*-Value
Sociodemographic variables				
Age	−0.023	0.531	0.017	0.683
Gender				
Female	Reference		Reference	
Male	−0.005	0.907	−0.056	0.173
Education	*F*(2) = 2.93	0.055	*F*(2) = 4.64	0.010
Low	Reference		Reference	
Middle	−0.009	0.854	0.000	0.993
High	0.085	0.073	0.130	0.011
Marital Status	*F*(2) = 1.08	0.341	*F*(2) = 0.090	0.490
Single/divorced	Reference		Reference	
Married/partner	0.126	0.191	0.012	0.878
Widowed	0.060	0.287	0.068	0.215
Living Situation				
Alone	Reference			
With others	−0.119	0.191	0.073	0.306
Psychosocial variables				
Perceived worries about the virus	0.081	0.068	−0.008	0.867
Perceived threat due to preexisting conditions	0.043	0.330	0.090	0.038
Being supportive of governmental measures	−0.047	0.169	−0.054	0.230
Perceived social support	−0.269	<0.001	−0.107	0.015
Resilience	−0.267	<0.001	−0.368	<0.001
Method factor	−0.105	0.102	−0.059	0.331
*R^2^*	0.257		0.225	
*n*	553		552	

Notes. Parameter estimation was done using the HC-3 method for robust standard errors. Omnibus F-tests were performed for all categorical variables.

**Table 3:** Multivariate regression model predicting depression and anxiety in a representative sample during the COVID-19 pandemic.

	Depressive Symptoms		Anxiety Symptoms	
	*β*	*p*-Value	*β*	*p*-Value
Sociodemographic variables				
Age	−0.057	0.163	0.008	0.868
Gender				
Female	Reference		Reference	
Male	0.079	0.045	0.010	0.808
Education	*F*(2) = 0.22	0.800	*F*(2) = 3.50	0.031
Low	Reference		Reference	
Middle	0.003	0.950	−0.038	0.451
High	0.024	0.590	0.073	0.147
Marital Status	*F*(2) = 4.80	0.009	*F*(2) = 0.06	0.943
Single/divorced	Reference		Reference	
Married/partner	−0.062	0.404	−0.011	0.885
Widowed	0.141	0.017	0.011	0.831
Living Situation				
Alone	Reference		Reference	
With others	−0.006	0.931	0.024	0.720
Psychosocial variables				
Perceived worries about the virus	0.078	0.090	0.121	0.005
Perceived threat due to preexisting conditions	−0.019	0.624	−0.007	0.852
Being supportive of governmental measures	−0.098	0.064	−0.060	0.182
Perceived social support	−0.220	<0.001	−0.084	0.067
Resilience	−0.185	<0.001	−0.197	<0.001
Method factor	−0.181	0.002	−0.188	<0.001
*R^2^*	0.235		0.179	
*n*	575		576	

Notes. Parameter estimation was done using the HC-3 method for robust standard errors. Omnibus F-tests were performed for all categorical variables.

## Data Availability

The data set used and analyzed during the current study is available from the corresponding author upon reasonable request.
